# Lethality of suicidal organophosphorus poisoning in an Indian population: exploring preventability

**DOI:** 10.1186/1744-859X-5-17

**Published:** 2006-11-21

**Authors:** Nilamadhab Kar

**Affiliations:** 1 Wolverhampton City Primary Care Trust, Corner House Resource Centre, 300 Dunstall Road, Wolverhampton, WV6 0NZ, UK

## Abstract

**Background:**

Suicide by organophosphorus poisoning is common in India. Study of factors associated with lethality may suggest methods for prevention.

**Methods:**

Severity of symptoms, biochemical manifestation of poisoning, degree of lethality and the outcome were studied with an aim to explore the modifiable factors associated with lethality and to discuss preventability. Clinical variables were collected; symptoms were rated by the physicians using global impression of severity; and the lethality was assessed by scale for assessment of lethality of suicide attempt (SALSA), in 100 consecutive patients with suicidal organophosphorus poisoning attending a medical college hospital in South India.

**Results:**

Fatal outcome (n = 26) was significantly associated with higher mean age, lower mean pseudocholinesterase level, longer duration between organophosphorus compound ingestion and specific intervention. All those who died had respiratory failure. Physicians' assessment of symptom severity and lethality as assessed by the SALSA could differentiate those succumbed and survived in a significant proportion.

**Conclusion:**

Majority of cases of organophosphorus poisoning were associated with severe symptoms and higher lethality. Intervention facilities decreasing the period between the ingestion of poison and initiation of treatment might prevent many deaths. Measures like restricting availability and banning more toxic organophosphorus compounds may help.

## Background

Poisoning is a common method of suicide, especially in the developing world [1]. In many Indian reports, the rates of poisoning as suicidal method range from 20.6% (10.3% organophosphorus) [2] to 56.3% (43.8% organophosphorus) [3,4]. It has remained so for almost a century, 44.2% in 1872-76 and 49.2% in 1972 [5]. Reported poisoning rates in the suicide attempters who attend hospital varies from around 40% to over 80% [6-8] in many Indian studies. Organophosphorus compounds available as pesticides are amongst the most common poisons used [3,9-12]. In hospital based studies mortality rates associated with pesticides have been reported up to as high as 50–70% [13]. Considerable proportions of children and adolescent attempters, 50% males and 60% females in one study [3], have used this method of attempt. Study of organophosphorus poisoning as a method of suicide attempt, its presentation in hospitals, lethality and outcome following intervention may provide insight for preventing death in a proportion of attempters.

Specific aim of this study was to evaluate suicide attempt by organophosphorus poisoning in an Indian patient population as they present to a hospital, severity of symptoms as observed by the physicians, biochemical manifestation of poisoning, the lethality of the suicide attempt, and the outcome. It also aimed to discuss the possible preventive ways based on modifiable factors associated with lethality.

## Methods

In a prospective study, consecutive 100 patients with suicidal organophosphorus poisoning attending a medical college hospital in South India were evaluated. Patients with multiple methods of attempt, including multiple substances for poisoning and accidental poisoning were excluded. The informed consent was taken from the relatives and later from the patient if he/she survived the attempt. Age, gender, clinical variables like interval between ingestion of poison and specific intervention (pralidoxime (PAM) injection for this index study), respiratory failure, duration of ventilatory support and mortality were studied. Pseudocholinesterase level was recorded. Manifestations of poisoning were recorded as mild, moderate or severe by the treating physicians as a global impression of severity of the symptoms.

Lethality was considered as the possibility or degree to which any biological change that could have endangered the life of the patient if not rescued or resuscitated. Lethality was studied by global impression of lethality item of the scale for assessment of lethality of suicide attempt (SALSA) with 5-levels of lethality (degree of lethality ranged from subliminal to extremely high) [14]. The mode of attempt, rescuability, degree of help required, maximum severity of physical symptoms manifested and the level of medical intervention required were considered in deciding about the lethality.

The statistical tests were done by SPSS package. The categorical data were analyzed by using chi-square tests and the continuous variables were compared by two-tailed t-tests. Statistical significance was defined at the standard 0.05 level.

## Results

There were 68 males and 32 female attempters (male to female ratio 2.1:1). Mean age and standard deviation (SD) of male (31.5 ± 12.37 years) and female (29.7 ± 15.62 years) attempters were not significantly different (t: 0.55, df: 98, p: 0.58). More women (90.6%) in contrast to men (70.6%) were married (χ^2^: 4.93, df: 1, p: 0.026).

Out of the 100 attempters 26 died. Nineteen males (27.9%) and 7 females (21.9%) succumbed to their attempt (ratio: 2.7:1); the difference between the proportions was not statistically significant. There was no significant difference between the genders regarding the clinical variables studied.

Comparisons of clinical variables of attempters who died with those survived their attempt are given in table 1. Mean age of the attempters who died was significantly more than that of who survived. Fatal outcome was significantly associated with lower mean pseudocholinesterase level which indicated probability of higher toxicity of ingested substance besides other factors. The duration of organophosphorus compound ingestion and specific intervention was significantly more in those died. The delay was due to many factors including lack of intervention facility locally and the time for travel. All the persons who died had respiratory failure, compared to 51.4% of the survived.

**Table 1:** Clinical variables associated with mortality following suicidal organophosphorus poisoning

Variables	n	Succumbed (n = 26)		Survived (n = 74)	
		n	%	n	%

Gender					
Male	68	19	27.9	49	72.1
Female	32	7	21.9	25	78.1
Age group in years					
<18	5	0	0.0	5	100.0
18–20	17	0	0.0	17	100.0
21–30	42	12	28.6	30	71.4
31–40	20	8	40.0	12	60.0
41–50	6	2	33.3	4	66.7
51+	10	4	40.0	6	60.0
Mean Age (SD) ^a^		35.88	(13.17)	29.02	(13.2)
Mean pseudocholinesterase level (SD) ^b^		1448.9	(1120.9)	3165.5	(2715.2)
Respiratory failure ^c^	64	26	40.6	38	59.4
Mean ventilatory duration in hours (SD)		7.15	(6.01)	5.17	(7.46)
Symptoms of poisoning ^d^					
Mild	8	0	0.0	8	100.0
Moderate	53	11	20.8	42	79.2
Severe	39	15	38.5	24	61.5
Lethality ^e^					
Subliminal	20	0	0.0	20	100.0
Low	15	0	0.0	15	100.0
Moderate	28	6	21.4	22	78.6
High	21	8	38.1	13	61.9
Extremely high	16	12	75.0	4	25.0
Mean PAM interval in hours (SD) ^f^		7.24	(6.28)	4.37	(4.63)

^a ^t: 2.28, df: 98, p: 0.024; ^b ^t: -3.12, df: 98, p: 0.002; ^c ^χ^2^: 19.76, df: 1, p: 0.00001; ^d ^χ^2^: 6.71, df: 2, p: 0.034; ^e ^χ^2^: 34.16, df: 4, p: 0.00000; ^f ^t: 2.47, df: 98, p: 0.015; Percentages are from the number of patients in that category of variable; figures in parentheses are SD.

Severity of symptoms and degree of lethality of considerable proportion of attempts were high. Global impression of severity of symptoms suggested that amongst those who died 42.3% had moderate and 57.7% had severe symptoms of poisoning in contrast to 10.8% mild, 56.8% moderate and 32.4% severe symptoms of those who survived (χ^2^: 6.7, df: 2, p < 0.05). Even though 61.5% with severe symptoms did survive, impression of the physicians regarding severity of symptoms significantly differentiated the outcome. Evaluation of lethality through SALSA suggested a trend with higher lethality indicator being associated with higher proportion of death in the attempters; for example, 75% of the attempters with extremely high lethality died compared to 38.1% with high and 21.4% of the moderate lethality (χ^2^: 34.2, df:1, p < 0.000).

## Discussion

The study tried to evaluate lethality in organophosphorus poisoning and to explore preventability. As reported in many previous studies males were more represented in the suicidal patients seen in a hospital setting [15]. A larger proportion of them compared to females died, but the difference was statistically not significant. Male/female ratio in the committers (2.7.1) is wider than the overall national ratio of 1.4:1 in suicide [1]; suggesting that in organophosphorus poisoning there is considerable preponderance of males in hospital setting. It might be relatively easier for men, some as farmers, to avail pesticides to use as suicide method as observed in other studies [3]. Concerns regarding increased suicide by farmers in India are well known [16,17].

Alarmingly 22% of attempters were 20 years old or less (17% within the age of 18 to 20 years); however all of them survived. It supports the reports that adolescents are a major risk group for suicide attempt [18]. Proportions of attempters who died were more in age groups of 31–40 years and 51 years and above, suggesting probability of higher risk of suicidal death in these age ranges. Mean age of those who died was significantly more than that of survivors.

Mean pseudocholinesterase level was significantly less in those who died compared to survivors. Though there are controversies about the correlation between plasma cholinesterase activity and the severity of organophosphorus poisoning [19], it is a marker of the organophosphorus intoxication. Number of hours passed between ingestion of poison and initiation of specific treatment had significantly influence on the outcome. It was significantly more in those who died. Respiratory failure which was observed in all who died and in 51.4% of survivors indicated the severity of the attempts and was one of the significant indicators for outcome.

Lethality is an important clinical variable for both medical and psychiatric evaluation and management. In contrast to intent-to-die which is a subjective measure, lethality is objective, more descriptive of the behaviour, and often correlate with the degree of intent [20]. In the index study findings indicated that organophosphorus poisoning in most attempters was clinically severe with higher degree of lethality. Physicians' assessment of symptom severity and lethality as assessed by the SALSA were able to categorize attempts into different grades of severity and lethality which could differentiate the outcome in a significant proportion.

It is presumable that severity of symptoms and toxicity of the compounds are related. In the index study most patients had moderate to severe symptoms, moderate to extremely high lethality and respiratory failure, besides significantly lower pseudocholinesterase level in those succumbed to the poisoning. Though toxicity of ingested substances was not directly studied; and many factors contribute to severity of poisoning, the above observations indirectly suggest probability of higher toxicity of ingested substances in most cases. Banning of extremely toxic pesticides and restriction of their use have been urged by World Health Organisation [13]. Restriction of availability of suicide methods has received some attention as a possible way of suicide prevention [4,21,22], though it is also reported that when one method is restricted then the suicidal methods change [4]. Legislation on drug availability and packaging has been effective elsewhere [13,22]. In addition, involving another individual from farmer community in the sale, use and safe disposal of the remaining content of organophosphorus compounds may help. Public education in this regard may be needed.

Efforts to minimize the period between ingestion of poison and initiation of specific treatment may help to decrease the chance of death in some. Most part of the duration from ingestion of poisoning to initiation of treatment was spent travelling/arranging transport to the hospital. In the developing world many attempters are referred to secondary and tertiary centres for lack of facility locally. It is a common observation that many attempters are brought dead to hospital [8]. Delay in arriving in hospital with facilities seriously curtails their effectiveness. Availability of basic facilities for treatment of organophosphorus poisoning at primary health centres (PHC) and local hospitals may change this negative outcome for many, if not for all. Increasing the ability of the primary care facilities to manage the medical complication of suicide attempt is a recognized intervention in China [21]. Periodic training to the doctors and other health care staff in community, improving their skills in assessment and management may help in dealing with more cases in community effectively in time before the duration of ingestion and specific intervention gets prolonged. It may help to have clear protocol and guidelines available for managing poisoning cases. The facilities for initiating treatment sooner and respiratory support locally and while transferring the patient (if needed) may increase the chance of survival for many attempters. However these suggestions would require further focused study for their effectiveness.

There are few limitations of the study. The sample was taken from a tertiary level of health care system. The findings in community sample attending primary or secondary centres may be different. Variables which may be clinically relevant regarding outcome such as existing other morbidities and treatments were not studied.

## Conclusion

In the index study a typical attempter who died was around 35 years of age, male, with low pseudocholinesterase level, respiratory failure, moderate to severe symptoms, moderate to extremely high lethality and an average duration of around seven hours from ingestion to specific intervention. Assessments of severity and lethality of the attempts were able to differentiate the attempters from the committers in most cases. Suicide prevention is a much broader multi-agency issue; and emphasis is given to preventing the act of attempt itself. However efforts to decrease the period between the ingestion and initiation of treatment, restricting availability of organophosphorus compounds, and banning more toxic ones may prevent some suicidal deaths following organophosphorus poisoning. Organophosphorus poisoning being a very common method of suicide attempt in developing world deserves specific attention.

## Competing interests

The author(s) declares that they have no competing interests.

## Authors' contributions

NK conceptualized, analyzed and interpreted data, and wrote the paper.

## References

[B1] Vijayakumar L (2004). Suicide prevention: the urgent need in developing countries. World Psychiatry.

[B2] Ponnudurai R, Heyakar J (1980). Suicide in Madras. Indian Journal of Psychiatry.

[B3] Gururaj G, Isaac MK (2001). Epidemiology of suicide in Bangalore.

[B4] Nandi DN, Mukherjee SP, Banerjee G, Ghosh A, Boral GC, Chowdhury A, Bose J (1979). Is suicide preventable by restricting the availability of lethal agents? A rural survey of West Bengal. Indian Journal of Psychiatry.

[B5] Nandi DN, Banerjee G, Boral GC (1978). Suicide in West Bengal – A century apart. Indian Journal of Psychiatry.

[B6] Badrinarayana A (1977). Suicide attempt in Gulbarga. Indian Journal of Psychiatry.

[B7] Kar N (1996). Psychosocial aspects of suicide attempt. MD Thesis, Utkal University, Bhubaneswar.

[B8] Arun M (2002). A comparative analysis of suicide and parasuicide. MD thesis, MAHE, Manipal.

[B9] Chugh SN, Aggarwal N, Dabla S, Chhabra B (2005). Comparative evaluation of "Atropine Alone" and "Atropine with Pralidoxime (PAM)" in the management of organophosphorus poisoning. Journal of Indian Academy of Clinical Medicine.

[B10] Rao CHS, Venkateswarlu V, Surender T, Eddleston M, Buckley NA (2005). Pesticide poisoning in south India: opportunities for prevention and improved medical management. Tropical Medicine and International Health.

[B11] Eddleston M, Szinicz L, Eyer P, Buckley N (2002). Oximes in acute organophosphorus pesticide poisoning: a systematic review of clinical trials. J Med.

[B12] Latha KS, Bhat SM (1999). Suicide attempts among youth: Correlates of medical lethality. Behavioural Medicine Journal.

[B13] Wadia RS (2003). Treatment of organophosphate poisoning. Indian J Crit Care Med.

[B14] Kar N (2002). Scale for Assessment of Lethality of Suicide Attempt.

[B15] Arun M, Yoganarasimha K, Palimar V, Kar N, Mohanty M (2004). Parasuicide – an approach to the profile of victims. Journal of Indian Academy of Forensic Medicine.

[B16] Venugopal D, Jagadisha (2000). An Indian perspective of farmer stress – a priority area for future research. International Journal of Social Psychiatry.

[B17] Sunder M (1999). Suicide in farmers in India. British Journal of Psychiatry.

[B18] Kar N, Pany M, Mishra BN, Sengupta J, Das I (1996). Risk factors of adolescent suicide attempt. Journal of Eastern Zonal Branch of Indian Psychiatric Society.

[B19] Nouira S, Abroug F, Elatrous S, Boujdaria R, Bouchoucha S (1994). Prognostic value of serum cholinesterase in organophosphate poisoning. Chest.

[B20] Hamdi E, Amin Y, Matter T (1991). Clinical correlates of intent in attempted suicide. Acta Psychiatr Scand.

[B21] Phillips M (2004). Suicide prevention in developing countries: where should we start?. World Psychiatry.

[B22] Gunnell D, Frankel S (1994). Prevention of suicide: Aspirations and evidence. BMJ.

